# Sensing of luminal contents and downstream modulation of GI function

**DOI:** 10.1002/jgh3.13083

**Published:** 2024-05-22

**Authors:** Kiran Devi Dontamsetti, Laura Camila Pedrosa‐Suarez, Rubina Aktar, Madusha Peiris

**Affiliations:** ^1^ Centre for Neuroscience, Surgery & Trauma, Blizard Institute, Barts and The London School of Medicine and Dentistry Queen Mary University of London London UK

**Keywords:** bacterial metabolites, brain–gut axis, enteric nervous system, nutrient sensing

## Abstract

The luminal environment is rich in macronutrients coming from our diet and resident microbial populations including their metabolites. Together, they have the capacity to modulate unique cell surface receptors, known as G‐protein coupled receptors (GPCRs). Along the entire length of the gut epithelium, enteroendocrine cells express GPCRs to interact with luminal contents, such as GPR93 and the calcium sensing receptor to sense proteins, FFA2 and GPR84 to sense fatty acids, and SGLT1 and T1R to sense carbohydrates. Nutrient–receptor interaction causes the release of hormonal stores such as glucagon‐like peptide 1, peptide YY, and cholecystokinin, which further regulate gut function. Existing data show the role of luminal components and microbial fermentation products on gut function. However, there is a lack of understanding in the mechanistic interactions between diet‐derived luminal components and microbial products and nutrient‐sensing receptors and downstream gastrointestinal modulation. This review summarizes current knowledge on various luminal components and describes in detail the range of nutrients and metabolites and their interaction with nutrient receptors in the gut epithelium and the emerging impact on immune cells.

## Graphical abstract

Overview of sensing luminal contents from diet and microbial by‐products across the GI tract. This results in changes to GI physiology including appetite regulation, glucose metabolism, gastric emptying and colonic motility.

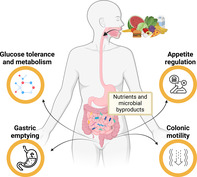

## Introduction

The mucosal surface of the gut epithelium is greater than that of the skin covering the whole body and is the primary site that is exposed to and interacts with the external environment. Unlike other peripheral tissues, the gastrointestinal (GI) tract is continuously exposed to a microbiota‐rich and changing chemical environment. In addition, the gut epithelium is modified by dietary components from ingested food and the variety of replicating microorganisms. The magnitude of these interactions is critical in regulating gut function, and everyone exhibits a unique personal signature. To respond to the dynamic, complex, and changing environment, the GI tract has developed sensing mechanisms to respond appropriately to the luminal contents.^1^ Gut cells are the first point of contact from ingested food and microbial components, and therefore have evolved to become specialized based on the gut region and its environment. Luminal sensing initiates from enteroendocrine cells (EECs) that are scattered throughout the gut epithelium and classified based on their contents and hormonal secretions.^2^ These cells are critical in the regulation of food intake, energy expenditure, glucose homeostasis, and a variety of metabolic functions from dietary components.^3^, ^4^ EECs express unique cell‐surface receptors that sense a broad range of signals. The complex assembly of nutrient receptors and chemical transmitters makes EECs ideal sensory cells in the gut with the specialized ability to detect luminal contents and convey these signals to the body by secreting hormones and neurotransmitters to communicate with the central and enteric nervous systems. This review focuses on the relationship between the luminal contents including bacterial metabolites, their interaction with the gut epithelium, and subsequent modulation of gut physiology.

### Role of EECs in luminal sensing and their downstream effects

EECs, which are found along the entire length of the GI tract, are critical sensors of the luminal environment. Both dietary nutrients and microbial fermentation products sensed by EECs can regulate the activity of these cells, which, in turn, have several downstream effects relevant to gut physiology and function. EECs are abundant throughout the GI tract but are scarcely expressed, accounting for <1% of cells in gut epithelium.^2^, ^5^ EECs are often characterized and distinguished by their cellular morphology, which is dependent on their relative position within the GI mucosa. EEC morphology can be either closed type, which does not directly interact with luminal contents, or open type with apical microvilli that extend to the lumen to sense and interact with luminal contents.^6^ Within the human small intestine and colon, EECs are open type in comparison to the stomach where EECs are typically closed type.^6^ The distribution of open‐type EECs is much higher in the regions where luminal exposure to nutrient‐derived metabolites is maximum (proximally), further supporting the role of EECs in the modulation of GI function in response to these nutrient ligands. EECs within the proximal gut release hormonal signals in response to nutrient absorption.^6^

There are several types of EECs, which are distinguished by the hormones and/or peptides they store and secrete (Table 1). EECs interact with the luminal contents via the nutrient‐sensing receptors they express, typically G‐protein coupled receptors (GPCRs), which have varying binding affinities to specific groups of nutrients such as proteins and fatty acids.^6^ Binding of nutrients to GPCRs results in cell activation and the release of hormone and peptide content. Some of the most important hormones/peptides released are glucagon‐like peptide 1 (GLP‐1), peptide YY (PPY) secreted by L cells, and cholecystokinin (CCK) secreted by I cells, expressed across the GI tract (described in Table 1). The peripheral contributions of these GI hormone/peptides, following the sensing of luminal contents from dietary intake, are significant in the transmission of sensory signals. Additionally, the physiological effects of the hormones/peptides stored in EECs on GI tract include changes to motility, modulation of energy intake, glucose tolerance/metabolism, and gastric emptying.^6^

**Table 1:** Overview of gastrointestinal hormones and nutrient counterpart

Gut hormone/peptide	EEC type	Location within GIT	Biological effect on gastrointestinal function	Nutrient ligand	Studies
GLP‐1 (glucagon‐like peptide‐1)	L‐cell	Mucosa of ileum and colon	Slows gastric emptying Induces satiety Decreases postprandial glucose levels (incretin effect)	Amino acids SCFAs LCFAs Carbohydrates Bitter compounds Phytochemicals Microbial products	9, 10, 15, 16, 22, 26, 32, 36, 37, 38, 39, 41, 44, 47, 48, 53, 59, 60, 67, 68
GIP (gastric inhibitory peptide)	K‐cell	Mucosa of the duodenum and jejunum	Regulates post‐prandial insulin Promotes lipid storage Reduces stomach gastric acid secretion	LCFA	36, 38, 39
CCK (Cholecystokinin)	I‐cell	Mucosa of the duodenum and jejunum	Increases satiation Regulates appetite Reduces rate of gastric emptying Promotes bile release form gallbladder and secretes pancreatic enzyme	Amino acids LCFAs Bitter compounds Microbial products	10, 14, 15, 16, 19, 48, 52, 56
5‐HT (5‐ hydroxytryptamine)	Enterochromaffin cell	Mucosa of the duodenum and jejunum	Regulates intestinal motility Modulates secretion in the intestine/sensory signaling Regulates satiation	SCFAs MCFAs Phytochemicals	9, 15, 55
PYY (peptide YY)	L‐cell	Mucosa of ileum and colon	Reduces appetite Slows gastric emptying Decreases GI motility for ileal brake (optimized nutrient absorption)	Amino acids SCFAs MCFAs LCFAs Carbohydrates Phytochemicals Microbial products	9, 15, 24, 28, 32, 37, 47, 48, 57, 60, 68
Gastrin	G‐cell	Mucosa of stomach antrum and duodenum	Increases gut motility Increases acid secretion for food digestion	Amino acids	13, 14
Ghrelin	P/D1‐cells (X/A‐cells rodents)	Mucosa of stomach fundus, corpus and antrum	Stimulates food intake/ regulates apettite Aids adipogenesis Acts as astroprokinetic agent Releases growth hormone	Amino acids MCFAs	9, 13, 27, 34, 69

This table summarizes key gastrointestinal hormones/peptides, their corresponding cell types and relative expression within the gastrointestinal tract, the physiological effects or functions of the hormones on the gastrointestinal tract, and the nutrients that trigger their secretion. Detailed are the exact functions the respective gut hormones are able to elicit on the gastrointestinal tract following the sensing of luminal contents. GLP‐1, glucagon‐like peptide‐1; GIP, gastric inhibitory polypeptide; CCK, cholecystokinin; 5‐HT, 5‐hydroxytryptamine/serotonin; PYY, peptide YY; GIT, gastrointestinal tract.

In direct response to dietary nutrients and bacterial metabolites, EECs exert physiological effects via gut hormones and/or peptides, including appetite modulation by acting within the arcuate nucleus of the hypothalamus.^3^ These hormones/peptides secreted within the lamina propria can also act via the vagus nerve, leading to anorectic effects mediated by sensory cell bodies in the nodose ganglia of the nucleus of the tractus solitary (brainstem).^6^

### Amino acid‐sensing receptors

#### GPR93

Protein hydrolysates from foods rich in amino acid content, such as meat, casein, and soybeans, have been found to act as ligands on GPR93.^7^, ^8^ GPR93 has been shown to be expressed in the duodenum–jejunal mucosal layer of rats and on human EECs, including both L cells and enterochromaffin (EC) cells.^7^, ^9^ Increasing the expression of GPR93 is shown to increase peptone‐induced endogenous CCK mRNA levels via intestine‐specific nutrient‐dependent EEC responses.^10^ Protein hydrolysates have been shown to stimulate GLP‐1 and CCK release in the intestinal murine EEC secretin tumor cell line (STC‐1), while free, unbound amino acids and undigested proteins failed to do so.^10^ Peptones from meat hydrolysates and egg albumin were shown to stimulate GLP‐1 release in a dose‐dependent manner, both in vivo using the STC‐1 murine cell line and ex vivo with isolated perfused rat intestine.^10^ The proposed mechanism for this luminally derived nutrient response is via GPR93‐mediated changes to intracellular calcium, driving membrane depolarization and increased cAMP levels.

#### Calcium‐sensing receptor

L‐Amino acids, derived following the digestion of foods with high dietary peptide content such as egg albumin, meat, and potatoes, are potent ligands for the Ca^2+^‐sensing receptor (CaSR). L‐Amino acids are sensed in the gut following amino acid metabolism and couple to gastric acid and/or pancreatic enzyme release.^11^ CaSR is expressed on the basal, lateral, and apical membranes of epithelial cells located in small intestinal crypts and villus and basal endocrine cells of colonic crypts in rats, mice, and humans.^12^, ^13^ Within the human stomach, expression of CaSR has been found on antral G cells and D cells, whereas in rats, duodenal CCK‐secreting I cells express the receptor.^13^, ^14^ In situ hybridization and immunohistochemistry has shown CaSR expression in the myenteric plexus of small and large intestines, demonstrating that this receptor is found on both epithelial and neuronal cell types.^12^

Interestingly, CaSR was seen to colocalize with 5‐hydroxytryptamine (5‐HT)‐secreting EECs (91%), most abundantly in the human antrum, while this pattern was not seen in mice, suggesting a species difference in CaSR colocalization with functional gut hormones/peptides.^15^

**Figure 1:**
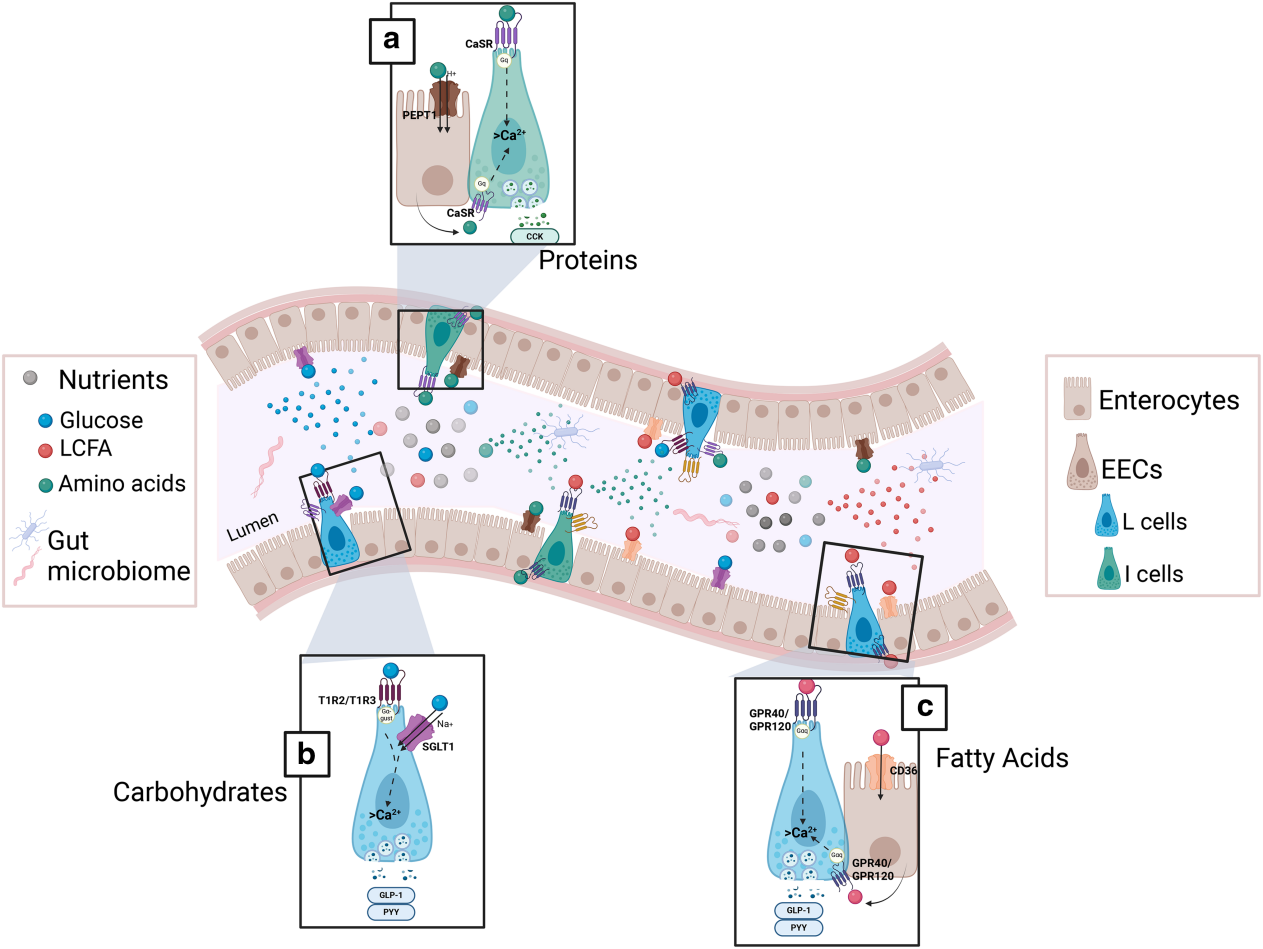
Nutrient luminal sensing in the gut. A schematic diagram of some of the sensing mechanisms of the main nutrients in the gut lumen by enterocytes and enteroendocrine cells (EECs) and the interaction with gut microbiota. (a) Protein sensing: small oligopeptides and amino acids are sensed mainly through Ca^2+^‐sensing receptor (CaSR) on the apical membrane of the EEC, which activates Gqα protein pathway and increases intracellular calcium levels to stimulate hormone secretion, mainly GLP‐1, PYY, and CCK. Other basolateral CaSR receptors can sense amino acids that are taken by enterocytes through PEPT1 in the lumen and transport them to the basolateral side. (b) Carbohydrate sensing: monosaccharides, mainly glucose, are sensed by apical receptors on the EECs, either through T1R2 or T1R3, which activate Gα–gustducin protein pathway or through SGLT1, which cotransports one molecule of glucose and one of sodium into the cell. Both mechanisms increase intracellular calcium and stimulate hormone secretion, mainly GLP‐1 and PYY. (c) Fatty acids sensing: long‐chain fatty acids (LCFAs) are sensed by EECs on the apical border predominantly by FFA1 and GPR120, which activate the Gq‐α protein pathway and increase intracellular calcium to stimulate hormone secretion, mainly GLP‐1 and PYY. Enterocytes are also capable of taking LCFAs through CD36 or by simple diffusion, in the form of triglycerides, and package them into chylomicrons to be transported to the basolateral side and sensed by other FFA1 or GPR120 receptors present on the basolateral membrane of EECs.

Activation of CaSR is shown to occur after nutrient absorption, that is, tryptophan and phenylalanine from dietary peptides, which stimulates the release of various hormones through the basolateral cell surface of CaSR.^16^, ^17^ CaSR specifically binds to calcium ions from the gut lumen at the orthosteric site, while aromatic and aliphatic polar L‐amino acids bind via the allosteric site.^5^, ^18^ The subsequent activation of CaSR results in the coupling to Gαq, as shown in Figure 1. Luminal calcium, L‐phenylalanine, peptone, and spermine all activate the CaSR expressed on G cells of wild‐type mice, resulting in the secretion of gastrin.^13^ Similarly, intestinal I cells respond to luminal calcium, tryptophan, and peptides by secreting CCK in wild‐type mice and cells,^19^ suggesting that CaSR is a nutrient sensor that modulates GI function. Additionally, administration of spermidine, a CaSR agonist, to rats results in reduced gastric emptying and intestinal motor activity.^20^

#### GPR6CA

GPRC6A is a receptor involved in metabolism, calcium homeostasis, and β‐cell activity, and is capable of binding basic amino acids derived from dietary protein.^17^ GPR6CA is expressed on rat gastric mucosal cells, specifically parietal, chief, and endocrine cells.^17^, ^19^ GPR6CA ligands include L‐lysine and L‐arginine, both of which have high binding affinity [17]. GPRC6A activation was shown to induce gastric acid and pepsinogen release in stomach cells isolated from Sprague–Dawley rats.^21^ When the uncarboxylated form of amino acid osteocalcin (GlaOC) is orally administered to wild‐type female mice, serum concentrations of GLP‐1 and glucose tolerance increases,^22^ whereas when GlaOC is orally administered to GPRC6A ^(−/−)^ male mice, there is glucose intolerance and increased incidence of high‐fat diet‐induced obesity.^22^, ^23^ Collectively, these data suggest an important role for GPRC6A in regulating metabolic and GI processes via amino acid stimulation.

### Short‐chain fatty acid (SCFA)‐sensing receptors

#### FFA2

FFA2 is a nutrient sensor that binds the short‐chain fatty acids (SCFAs) acetate, propionate, and butyrate derived from microbial fermentation of non‐digestible carbohydrates and couples to Gαi/o and Gαq.^24^, ^25^ FFA2 mRNA was identified in rat mucosa samples from the distal ileum and colon on PYY‐containing EECs, which is close to the site of SCFA production, and FFA2 was found on L cells in the human ascending colon.^15^, ^24^ Interestingly, Karaki *et al*. found FFA2 mRNA in human mucosal separations but not in muscle or submucosal layers.^26^ FFA2 has also been identified in white adipose tissue of mice and shown to be overexpressed in adipose tissue of mice when fed a high‐fat diet.^27^ Additionally, activation of FFA2 by non‐digestible and fermentable dietary starch and fiber increases GLP‐1 secretion, an observation further supported by data from FFA2 ^(−/−)^ mice where GLP‐1 levels are significantly lower than in wild‐type mice.^26^

#### FFA3

FFA3 is another SCFA‐sensing receptor critical for gut–brain communication, which shares the same ligands as mentioned above for FFA2.^28^, ^29^ FFA3 is expressed on human enterocytes, EECs, and autonomic and somatic sensory ganglia of mice.^30^, ^31^ In mice, FFA3 mRNA expression peaked in the distal jejunum, where 32% colocalized with CCK‐producing I cells^15^. Similarly, FFA3 mRNA was most abundant across the duodenum to the jejunum of humans.^15^ Moreover, there was a 69% overlap of cells stained individually with GLP‐1, PYY, and FFA3 in the human ascending colon.^15^ FFA3 activation is demonstrated to modulate GI tract functionality; specifically, FFA3‐deficient mice have decreased PYY secretion, which correlated with decreased absorption of SCFAs, leading to increased intestinal transit time and reduced energy efficiency. Binding of SCFAs, propionate, and butyrate to FFA3 expressed on peripheral nerves of rats resulted in improved glucose control and decreased adiposity.^28^ The evidence presented demonstrates the clear role of FFA3 as a nutrient‐sensing receptor where its activation leads to downstream GI modulation such as increased gut transit time, increased adiposity, and increased secretion of GLP‐1 in EECs.^32^

### Medium‐chain fatty acid (MCFA)‐sensing receptors

#### GPR84

High‐fat diets are rich in MCFAs, which act as endogenous ligands on GPR84.^33^ GPR84 is an orphan‐surface‐expressed GPCR that couples to Gi, and mRNA data show expression from antrum to rectum in both humans and mice, although the most abundant expression is in the small intestine of both species.^15^, ^34^ GPR84 mRNA expression appears responsive to diet because (i) mice fed a high‐fat diet (60% fat energy source) have higher mRNA levels compared to chow‐fed mice^9^, ^15^, ^27^, and (ii) reduced intake of high‐fat milk (containing >25% MCFAs) reduces mRNA expression.^9^, ^35^ GPR84 protein is expressed on X/A ghrelin cells and somatostatin‐secreting D cells in mice,^9^, ^27^, ^34^ as well as on L cells and EC cells in the human colon.^15^ Colocalization studies have shown a 33% co‐expression of GPR84 on 5‐HT‐containing cells and GPR120 receptors in human EC cells. GPR84 colocalization is much less on PYY‐containing L cells (10%).^9^

GPR84 can be stimulated via binding of dietary fatty acids with a carbon length of 9–14, including capric acid, undecanoic acid, and lauric acid, which bind with modest potency as shown by cAMP assays.^9^ Lauric acid binds to GPR84 in the human proximal colon to induce the release of the appetite‐regulating hormones PYY, GLP‐1, and 5‐HT.^9^, ^15^ Combining lauric acid and TUG‐891—a potent FFA4 agonist—has also been shown to increase colonic afferent firing in proximal mouse colon tissue.^9^ Taken together, GPR84 signaling is highly responsive to the dietary intake of MCFA‐rich food sources such as milk fats and coconut oil.

### Long‐chain fatty acid (LCFA)‐sensing receptors

#### FFA1

LCFAs from the diet affect energy metabolism and glucose homeostasis by binding to FFA1.^36^ FFA1 functions as a nutrient sensor for MCFAs/LCFAs and is expressed along the GI tract of both humans and mice. Specifically, it is most abundant on EECs of the duodenum and in the ileum of mice, while in humans FFA1 is highly expressed in the jejunum .^15^ FFA1 has also been identified on the taste buds of rats, as well as on the pancreatic β‐cells and brain in humans, specifically in the substantia nigra and medulla oblangata.^9^, ^36^

Both MCFAs and LCFAs were found to bind to FFA1, increasing insulin secretion without changing glucose metabolism in wild‐type mice fed a high‐fat diet.^37^ Synthetic agonists of FFA1 recruit Gα_q_ and Gα_s_, where Gα_s_ increases cellular levels of cAMP and leads to GLP‐1, gastric inhibitory polypeptide (GIP), and PYY secretion in adult male Wistar rats. When FFA1‐null mutant mice were administered a high‐fat diet, Edfalk found reduced plasma glucose levels as well as GIP and GLP‐1 secretion when compared to wild‐type mice.^36^, ^38^, ^39^ In in vitro and in vivo rodent models, CCK release was induced via sensing of dietary LCFAs binding to FFA1 expressed on duodenal I cells, but GLP‐1 secretion was not similarly induced in small intestinal studies in rats.^16^

#### FFA4

FFA4 is an LCFA receptor capable of binding to a range of LCFAs, although α‐linolenic acid is the most potent agonist,^16^, ^40^, ^41^ while docosahexaenoic acid C22:6 (n‐3) and eicosapentaenoic acid C20:5 (n‐3) also bind to and activate FFA4 in the micromolar concentration range.^41^, ^42^

FFA4 expression is localized primarily on L cells in the large intestine of humans (Fig. 1).^41^ FFA4 mRNA levels peaked in the proximal colon of mice, while in humans FFA4 mRNA increases along the length of the GI tract, with the highest expression in the rectum.^15^ A small proportion of FFA4 receptors are expressed on type II taste bud cells of rats.^37^ Additionally, FFA4 is found in the intestinal secretin tumor cell line (STC‐1), displaying similarities to native ECCs. In humans, FFA4 expression is strongly correlated with body mass index, as receptor expression is higher in biopsies from the stomachs of morbidly obese patients compared to lean patients.^43^

Activation of FFA4 evokes the Gαq/11‐coupled pathway and can induce GLP‐1 and PYY secretion. Martin *et al*. suggested that dietary lipids affect the perception thresholds of sucrose and fats through the activation of FFA4, leading to GLP‐1 secretion.^37^ While the molecular mechanism is not fully elucidated, the activation of FFA4 by selective LCFA agonists may induce GLP‐1 release from the taste cells of the mouse circumvallate papillae.^41^

Oral administration of corn oil (an endogenous ligand of both FFA1 and FFA4), which has a high LCFA content, was found to lead to GLP‐1 secretion in both wild‐type mice and FFA4 ^(−/−)^ mice. Characterization and cloning of rat FFA4 have demonstrated an opposite effect, where plasma GLP‐1 and insulin levels were significantly increased in comparison to octanoic acid. Peiris *et al*. have shown that FFA4 stimulation with TUG‐891, a more selective and potent agonist of FFA4, increases PYY and GLP‐1 secretion in human colonic L cells.^9^ Interestingly, co‐stimulation of FFA4 and GPR84 expressed on human colonic mucosal tissue in vitro resulted in the activation of two separate pathways, where GPR84 exclusively induced intracellular pERK while FFA4 activated pCAMKII. This leads to synergistic cell activation and release of GLP‐1 and PYY.^9^, ^15^

### Carbohydrate sensing

Complex carbohydrates are broken down by digestive enzymes into monosaccharides, such as glucose and fructose, and can be sensed by the apical surface of EECs.^5^, ^32^ Specifically, glucose is sensed via sweet taste receptor class 1 (T1R) or the sodium‐glucose cotransporter‐1 (SGLT1),^44^, ^45^ while fructose has GLUT5 as an apical receptor in the lumen and it is also associated with GLUT2 on the basolateral membrane.^32^ Surprisingly, recent studies using intestinal stem cells have shown evidence that besides EECs, paneth and goblet cells also utilize GLUT5 as a fructose‐sensing receptor.^46^

T1R1, T1R2, and T1R3 expression are shown in human and mouse mucosa of the stomach, small intestine, and colon, although T1R2 is absent in both stomach and colon.^15^ T1R2 and T1R3 colocalize on humans and mouse L cells and sense intraluminal glucose, triggering membrane depolarization leading to GLP‐1 and PYY release (Fig. 1),^47^ an action that is specifically inhibited in the presence of the T1R inhibitor.^32^

In primary human L cells, GLP‐1 release is mediated by SGLT1.^32^ In mouse, knockout of SGLT1 caused a reduction in the glucose‐dependent GLP‐1 detection levels.^32^ GLP‐1 release mediated by intraluminal glucose has been identified to increase insulin release, regulate energy intake, and suppress appetite.^32^ In recent clinical trials, glucose infusion to the ileum of healthy subjects increased GLP‐1 and PYY secretion, leading to reduced appetite.^48^ On the contrary, studies of obesity and high‐fat diets have shown reduced GLP‐1 levels, reduced expression of L cells and specific L‐cell genes such as SGLT1, and reduced satiation in response to carbohydrates infusion.^48^

### Bitter taste and phytochemicals

Some of the most known bitter tastants are chloroquine and quinine—both antimalaria and anti‐inflammatory drugs—and polyphenols, found in berries and grapes, among others.^49^ Studies in mice have shown that bitter tastants are sensed by receptors of the type 2 family (T2R), which activate α‐gustducin, a G‐protein subunit.^5^ In mice, cycloheximide and denatonium (DB) activated T2R5 and T2R8, respectively, while in humans, denatonium and 6‐*n*‐propyl‐2‐thiouracil activated T2R4.^5^ Expression of the T2R family receptors has been demonstrated in the stomach, small intestine, and colon of mice and rats.^50^ Specifically, studies in knock‐in mice have shown that goblet cells express T2R131 and T2R138, both of which colocalized with chromogranin A‐positive cells, a marker for EECs.^50^, ^51^ In hormone release studies, CCK secretion was associated with DB stimulation via calcium influx in rodents,^52^ while GLP‐1 secretion was observed with quinine and DB in mice and humans.^53^ The ingestion of a bitter compound is sensed by tongue taste buds as potentially toxic.^54^ Hence, EEC release of anorexigenic peptides in response to bitter compounds, and the expression of bitter taste receptors in mucus secreting cells, could be associated with a defense mechanism of the body to limit additional harmful ingestion.

Phytochemicals, such as spices found in mustard and chili peppers, have been found to stimulate the transient receptor potential cation channel 1 (TRPA1), leading to EEC hormone secretion.^21^ In a guinea pig, ileal EC released 5‐HT as a response to allyl isothiocyanate (AITC) (mustard) and cinnamaldehyde (cinnamon).^55^ In mouse duodenal mucosa, AITC stimulated CCK secretion via calcium influx^56^, and on mouse primary L cells TRPA1 activation induced GLP‐1 release.^44^ Yet, in a mice study with methyl syringate (honey) stimulation, PYY plasma levels were increased, while GLP‐1 levels were not.^57^ Moreover, in a more recent study in healthy human subjects, intraduodenal infusion of capsaicin (chili peppers) significantly increased satiety but was not able to improve GLP‐1 or PYY release, suggesting that satiety was rather due to symptoms of burning sensation, nausea, and pain.^58^ Thus, more studies of the association of phytochemicals with gut function regulation via EEC hormone release are needed.

### Microbial fermentation products

#### Bile acids

Bile acids, produced in the liver, are deconjugated by gut microbiota, principally by Bacteroides spp. and Lactobacillus, to be sensed by the G protein‐coupled bile acid receptor GPBAR1 (also known as TGR5) or by the farnesoid X receptor (FXR).^48^ The expression of TGR5 associated with bile acid stimulation has been found mostly on colonic L cells, specifically in the basolateral position.^59^ In fact, preclinical studies in mice have shown that TGR5 has a key role in GLP‐1 secretion and is capable of increasing insulin sensitivity and regulating energy intake.^59^ Moreover, clinical studies with rectal administration of taurocholic acid stimulated the secretion of GLP‐1 and PYY and increased fullness in a dose‐dependent manner.^60^ Likewise, FXR is mostly expressed in the liver and intestine, principally on enterocytes and L cells.^48^ However, on L cells, FXR stimulation decreased GLP‐1 secretion via glycolysis inhibition, which was reversed in FXR‐deficient mice.^48^ Considering studies that suggest there is a glucose‐dependent effect of bile acids in GLP‐1 secretion,^60^ in pathophysiological conditions, there could be a beneficial effect of induced FXR deficiency via the FXR/GLP‐1 pathway.

#### Microbial byproducts and metabolites

Within the gut lumen, gut microbiota secrete different metabolites which can act as ligands for nutrient receptors as described earlier in the case of SCFAs.^61^ Further examples of metabolites include indole, a product of tryptophan metabolism from *Escherichia coli* and *Proteus vulgaris*,^62^ and indole‐related compounds have been identified as ligands for GPR84 in humans.^63^ As mentioned in previous section, GPR84 is expressed from stomach to distal colon and has been associated with different types of EECs. In mouse, indole was associated with a dose‐dependent increase of GLP‐1 release from colonic L cells by increasing calcium entry, although no GPCR was associated with this effect.^44^ Other metabolites such as lipopolysaccharides (LPS)—a bacterial breakdown product also known as an endotoxin—increased plasma levels of GLP‐1 in both fasting and glucose challenge conditions on experimentally induced hyperglycemic mice.^44^ However, a chronic 6‐week administration of LPS at low doses, mimicking the chronic low‐dose increase of LPS in obese patients, was capable of inhibiting satiety and motility regulation induced by CCK release.^48^

Besides being an intermediate of the tricarboxylic acid cycle, succinate is also produced by microbiota as a byproduct of anaerobic fermentation.^64^ Germ‐free (GF) mice exhibit no detectable succinate levels in their feces, suggesting that the gut microbiome is a key source of luminal succinate,^65^ although succinate levels are usually low in the lumen as it is continuously converted as an intermediate of the propionate pathway.^64^ The succinate receptor SUCNR1 (also known as GPR91) is highly expressed during metabolic stress associated with elevated concentrations of extracellular succinate.^56^ In the intestinal epithelium, tuft cells utilize GPR91 for succinate sensing, mainly for initiating an immune response against bacterial and parasitic infections.^66^ Studies in EEC hormone release have shown that succinic acid, a fermentation product of alcoholic beverages, created a dose‐dependent CCK release from EEC cell line STC‐1 stimulation.^56^ GF mice colonized by the bacterium *Bacteroides thetaiotaomicron* (Bt) had a predominant increase of GLP‐1‐positive cells in the proximal colon, which was replicated by adding the major Bt metabolites acetate, propionate, and succinate.^67^ In fact, Bt conventionalization restored the expression of TLRs, predominantly TLR2, in GF mice and mediated the activation of L cells secreting GLP‐1 and PYY.^68^ However, for other hormones, succinate stimulation showed no effect on ghrelin secretion in the stomach.^69^ Thus, more studies to unravel the mechanism and sensing of luminal receptors of microbiome metabolites and endotoxins and their effect on hormone release are needed.

## Paracellular‐acting metabolites and effect on GIT function

Immune cells also interact with dietary nutrients via paracellular mechanisms to sense glucose, glutamine, and fatty acids.^70^ Nutrient‐sensing GPCRs are expressed on immune cells, such as FFA2, on neutrophils, which improves leukocyte migration and increases phagocytosis.^70^ Dendritic and mast cells express GPR91 to increase the production and migration of inflammatory cytokines such as TNFα and IL‐1β and regulate intestinal barrier function.^71^ Besides GPCRs, intestinal barrier function is strengthened by microbial fermentation products such as SCFAs, indole, and bile acids, which can increase the expression of paracellular junction proteins such as claudins, occluden‐1, and occludin.^48^

The association between nutrients and neutrophils has been widely studied via GPCRs, specifically for FPR1, FRP2, and, recently, FFA2 and GPR84.^72^ Both receptors FFA2 and GPR84 are involved in neutrophil degranulation, chemotactic migration, and production of NADPH oxidase‐derived reactive oxygen species (ROS) in human and mice studies.^73^ Likewise, GPR84 is also expressed on primary human monocytes, monocytes derived from macrophages, and macrophages.^72^

In inflammatory bowel disease (IBD), where there is significant immune activity, nutrient availability influences inflammatory pathways. In Crohn's disease, patients with enteral dietary fiber nutrition had increased FFA2 expression in polymorphs, which increased infiltration^74^, while surgical resection tissue had increased GPR91 expression compared to controls.^71^ Moreover, metabolomic studies of IBD patients showed increased succinate levels in mucosal lesions.^71^ In ulcerative colitis, GPR84 expression is upregulated in the human colon, and mucosal infiltrative macrophages express GPR84.^72^ Other GI diseases, such as eosinophilic esophagitis, are associated with SCFAs, where high butyrate levels drive increased FFA3 expression on Th2, leading to eosinophil accumulation.^75^ Thus, the role of nutrients as regulatory factors of immune cells is highly relevant for the understanding of pathways and to develop new therapies for GI diseases.

## Conclusion

The gut has evolved to detect the nutritional status of the body with precision via a complex, integrated network of neural and hormonal signals that sense the contents of the lumen. This gut connectome provides a pathway for bidirectional communication to the brain, which centrally processes neural information. The cells of the gut epithelium contain specialized cellular machinery required to sense and respond to luminal contents by a variety of receptors that modulate components of fatty acids, carbohydrates, amino acids, as well as bacterial metabolites. Although advances have been made in understanding how the gut senses components of the gut, further research is needed to completely elucidate the sensing mechanisms in these specialized cells and how specific hormones are released to modulate gut function. This is critical, as dietary manipulations that modulate receptor function can be targeted to treat metabolic diseases including obesity and type 2 diabetes, which pose a huge public health burden.
